# Salol

**Published:** 1886-10

**Authors:** 


### SALOL.

In a communication to the Medical Society of Berne, Dr. Sahli mentions the injurious effects upon the stomach, produced by the prolonged use of salicylate of soda. He addressed a note to Prof. Neucki, asking him if there was not some other preparation of salicylic acid, which would be free from this objection. Prof. Neucki, who had for three years been studying the chemical, physiological and antiseptic properties of *salicylate of phenol*, or *salol*, immediately recommended a trial of this drug to Dr. Sahli

Salol contains 38 per cent. of phenol, and appears in the form of a white crystalline powder. It has no distinct odor or taste, it is insoluble in water, but is readily soluble in alcohol. It is best administered in capsules or tablets with sugar of milk or licorice powder.

The experiments of Neucki have demonstrated that salol is decomposed by the pancreatic juice into a free acid and an alcohol, that is, into salicylic acid and phenol. It is eliminated by the kidneys, and it may be recovered in the urine in the form of sulphophenic and salicyluric acids.

With regard to the administration of the new product, Sahli has attained excellent results in cases of articular rheumatism, by giving fifty centigrams (gr. viiss) every twenty-four hours. The dose may, however, be increased to six or eight grams (ʒiss-ii) a day, without danger. This remedy does not exhaust the stomach, is almost tasteless, and rarely produces tinnitus, thus affording great advantages over salicylate of soda and salicylic acid. The urine, after the administration of salol becomes dark, resembling that of patients who have taken phenic acid.

Besides its use in articular rheumatism, Dr. Sahli believes that it will be valuable in the treatment of diabetes insipidus, phthisis and as an antipyretic. To consumptives he would recommend beginning with small doses.

Salol has been used with success in the treatment of urticaria, typhoid fever, miliary fever, intestinal catarrh, cholera, intestinal parasites, catarrh of the bladder, ozaena, otorrhœa, neuralgia and migraine.

Salol is also a powerful antiseptic. If it does not destroy microbes, it retards their development. It has been proposed to use it in all cases where sublimates and iodoform are now employed.—*Bull. Gen. de Therapeutique*, Aug. 15.

